# G Proteins and GPCRs in *C. elegans* Development: A Story of Mutual Infidelity

**DOI:** 10.3390/jdb6040028

**Published:** 2018-11-25

**Authors:** Daniel Matúš, Simone Prömel

**Affiliations:** Rudolf Schönheimer Institute of Biochemistry, Medical Faculty, Leipzig University, 04103 Leipzig, Germany; daniel.matus@medizin.uni-leipzig.de

**Keywords:** GPCRs, G proteins, development, receptor-independent function

## Abstract

Many vital processes during *C. elegans* development, especially the establishment and maintenance of cell polarity in embryogenesis, are controlled by complex signaling pathways. G protein-coupled receptors (GPCRs), such as the four Frizzled family Wnt receptors, are linchpins in regulating and orchestrating several of these mechanisms. However, despite being GPCRs, which usually couple to G proteins, these receptors do not seem to activate classical heterotrimeric G protein-mediated signaling cascades. The view on signaling during embryogenesis is further complicated by the fact that heterotrimeric G proteins do play essential roles in cell polarity during embryogenesis, but their activity is modulated in a predominantly GPCR-independent manner via G protein regulators such as GEFs GAPs and GDIs. Further, the triggered downstream effectors are not typical. Only very few GPCR-dependent and G protein-mediated signaling pathways have been unambiguously defined in this context. This unusual and highly intriguing concept of separating GPCR function and G-protein activity, which is not restricted to embryogenesis in *C. elegans* but can also be found in other organisms, allows for essential and multi-faceted ways of regulating cellular communication and response. Although its relevance cannot be debated, its impact is still poorly discussed, and *C. elegans* is an ideal model to understand the underlying principles.

## 1. GPCRs and G Proteins—Together and Apart

**Figure 1:**
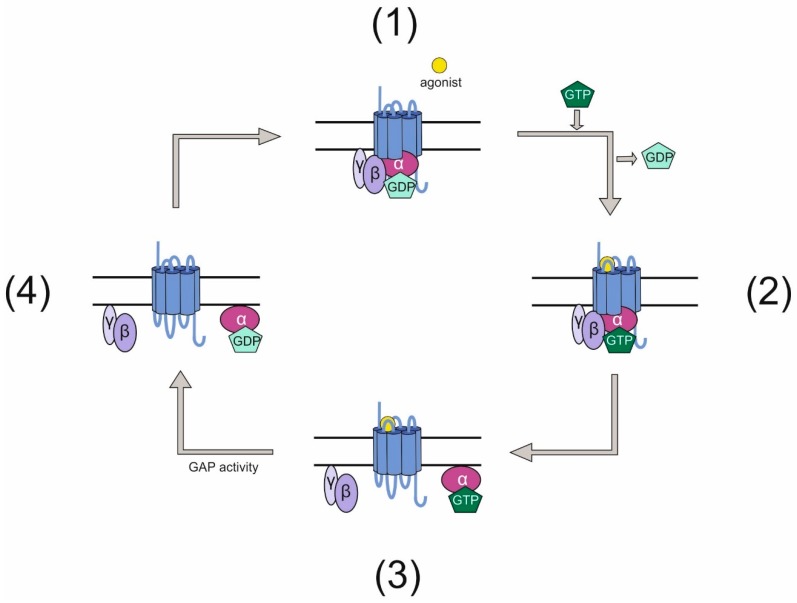
Classical G-protein cycle. (1) An inactive GPCR is associated intracellularly with a heterotrimeric G protein. This G protein has bound GDP on its Gα subunit. (2) Upon activation of the GPCR by an agonist, the receptor acts as a GEF for the G protein and exchanges GDP for GTP. (3) The active G protein dissociates into Gα and a Gβγ complex, both of which can mediate signals. (4) After signals have been elicited, the GTP bound to Gα is hydrolyzed rendering the G protein inactive. The G protein subunits re-associate and bind the GPCR.

G protein-coupled receptors (GPCRs) and heterotrimeric G proteins are molecules, which together form one of the key systems essential to transduce extracellular cues into cells and mediate signals. Thereby, GPCRs are seven transmembrane receptors binding the G proteins with their intracellular parts. These G proteins are trimers composed of α, β, and γ subunits with the α subunit being able to bind the guanine nucleotide GDP when attached to the receptor. Upon receiving a stimulus such as photons, hormones, proteins, or peptides, the GPCR acts as a guanine nucleotide exchange factor (GEF) exchanging the GDP bound to the Gα for a GTP. This activates the G protein, which is then able to mediate signals by both the Gα subunit and the dissociated Gβγ complex. Subsequently, the GTP is subject to hydrolysis, thereby inactivating the G protein, which re-associates as a trimer with the GPCR and thus completing this so-called G-protein cycle (Figure 1).

In the last few decades, many studies have shown that the classical interaction between G proteins and the corresponding receptor is not the only way these molecules can signal. For GPCRs, an increasing body of data suggests that they are able to signal completely independently of G proteins, for instance via arrestins (reviewed in [1]) or Jak-Kinases [2]. Similarly, heterotrimeric G proteins can function in the absence of GPCRs [3,4]. These findings paved the way for the notion that GPCRs and G proteins can fulfill a plethora of functional roles beyond classical signaling. However, many details of the mechanisms and the relevance of G protein-independent GPCR signaling and GPCR-independent G-protein signaling remain poorly understood. 

## 2. GPCRs and G Proteins Are Essential for *C. elegans* Development

As GPCRs and G proteins are involved in many physiological processes and are vital in health and disease, it is not surprising that they are also found to be linchpins for orchestrating signaling processes during development in many organisms. The *Caenorhabditis elegans* genome encodes for more than 1000 GPCRs [5] and its heterotrimeric G protein repertoire comprises 21 Gα subunits, 2 Gβ and 2 Gγ proteins [6]. Throughout *C. elegans* development and especially during early embryogenesis, several essential processes are governed by GPCRs and/or G proteins, together or separately. This offers an ideal and well-characterized system to gain insights into the mechanisms underlying the diverse functions of GPCRs and G proteins as well as their fundamental impact in biology. Although certainly not all processes, in which GPCRs/G proteins have a function, are fully uncovered yet, several examples highlight the potential of the system.

### 2.1. Gα Subunits Dictate Asymmetric Spindle Postitioning

**Figure 2:**
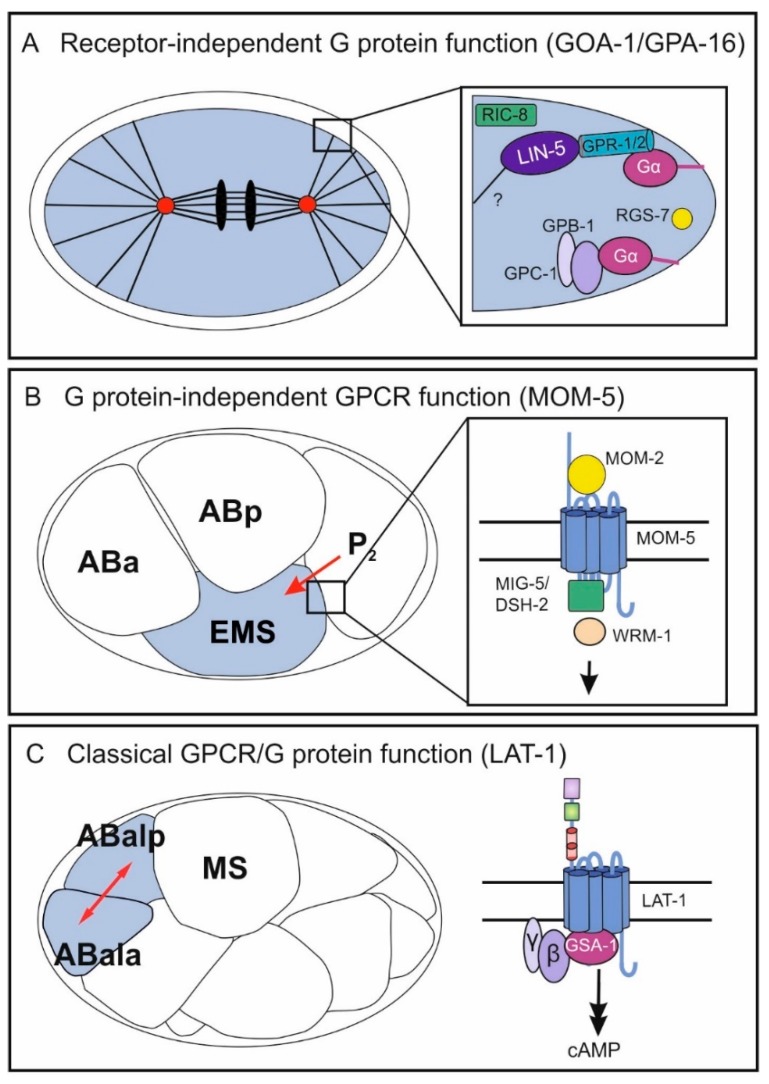
Independent and classical functions of GPCRs/G proteins in different processes during *C. elegans* embryonic development. In the early embryo, all three mechanisms occur in different biological contexts: the receptor-independent G-protein function (GOA-1/GPA-16) (**A**), the G protein-independent GPCR function (MOM-5) (**B**), and the classical GPCR/G protein pathway (**C**). (**A**) GOA-1 and GPA-1 control pulling force on astral microtubules leading to two asymmetric cells after the first division. The Gα proteins function in a ternary complex with GPR-1/GPR-2 and LIN-5 and are modulated by the GEF RIC-8 and the RGS RGS-7. Further, the Gβ and Gγ subunits GPB-1 and GPC-1 are hypothesized to contribute to regulation of pulling forces. (**B**) The Frizzled class GPCR MOM-5 mediates a signal from the P_2_ blastomere polarizing the neighboring EMS cell. Upon activation by the Wnt ligand MOM-2, MOM-5 activates the disheveled homologs MIG-5/DSH-2 and subsequently involves the β-catenin WRM-1. Note that asymmetric localization of the different components is not depicted. (**C**) A classical GPCR/G protein pathway in embryonic development is realized by the Adhesion GPCR LAT-1, which controls anterior–posterior cell division of several blastomeres including ABal. LAT-1 activates the Gs pathway via the Gα protein GSA-1 yielding an increase in the second messenger cAMP. For the physiological relevance of each GPCR/G-protein function, see Section 1. Mechanistic details are discussed in Section 2 and Section 3.

Already during the first cell division, G proteins are involved in asymmetric positioning of the mitotic spindle in the one-cell-stage embryo to ultimately promote asymmetric cell division. The two maternally required Gα subunits GOA-1 and GPA-16 are in part redundantly required for transducing polarity cues to generate pulling force on the mitotic spindle during cell cleavage [7,8] (Figure 2A). While GOA-1 is a Gα_o_ subunit, homology comparison of the structurally similar GPA-16 with mammalian Gα subunits does not allow for its clear identification as a Gα_o_ protein [6]. In wild-type embryos prior to the first cell cleavage, centrosomes align along the anterior–posterior axis by a rotation during prophase [9] and pulling forces are exerted along this axis. These are asymmetric in the way that the posterior spindle pole moves closer to the cortex than the anterior pole [10,11], subsequently leading to the generation of two cells of unequal size. Embryos mutant for *gpa-16* exhibit a reduced and more symmetric pulling force during cell cleavage [12]. Similarly, loss of function of *goa-1* and *gpa-16* causes a loss of nuclear rotation as well as reduced pulling of the spindle toward the posterior [7,8] yielding two symmetric cells. Both G proteins exert a pulling force on astral microtubules in a ternary complex comprising the GoLoco motif-containing GPR-1 or GPR-2 and the coiled-coil protein LIN-5 [7,8,13,14,15]. Through its myristoylation, the Gα protein anchors the complex to the plasma membrane and in parallel binds GPR-1/2, which also associates with LIN-5 [15,16,17,18]. How the asymmetric pulling is subsequently realized is not entirely understood, but several studies show the involvement of microtubules and dynein in this process [17,19,20]. Dynein appears to serve as a connecting molecule between the ternary complex and astral microtubules [21]. However, whether it is the core force generator is still debated. Interestingly, the polarity cue triggering the asymmetric spindle pulling is not elicited by a GPCR but rather by cortically localized cytoplasmic PAR proteins (reviewed in [22]) and modulated mostly via regulators of G protein signaling (RGS) such as RGS-7, guanine-nucleotide dissociation inhibitors (GDIs) [8], and GEF proteins such as RIC-8 [23]. The molecular details of G-protein activation involving these modulators and the mechanisms behind its function will be discussed in Section 3.

Not only the Gα subunits but also the associated Gβ and Gγ proteins have been identified to be involved in spindle positioning and pulling force generation. Both β subunits present in *C. elegans* are expressed in embryos [24,25], but only GPB-1 seems to be required for spindle positioning [7,25], functioning with the Gγ subunit GPC-2 [7]. The distinct roles of Gβ and Gγ remain elusive to a certain extent. It has been postulated that one function of GPB-1 in this context might be the regulation of Gα availability by forming a heterotrimer as the amount of unbound α subunits influences pulling forces. Deficiency of GPB-1 or GPC-2 increases pulling forces toward the anterior and elevates rotational movement [8,23,26], suggesting that the Gβγ complex is negatively regulating force generation. In line with these data, its amount and distribution within the cell is asymmetric and dynamic during cell division [27]. 

Several lines of evidence show that positioning of mitotic spindles and dictating pulling force during later cell divisions are also likely to be guided by the G proteins GOA-1 and GPA-16. In embryos lacking both Gα subunits, the nucleus in the P_1_ blastomere does not rotate, and mitotic spindle directionality does not form correctly [7]. Further, embryos contain polyploid nuclei and fewer cells [15]. A second line of evidence using a temperature-sensitive *gpa-16* mutant highlights that spindle orientation is defective in the EMS blastomere when shifting mutant embryos to the restrictive temperature after the second cell division [8]. However, this effect is not fully penetrant, most likely due to the partially redundant signaling of the MES1/SRC-1 pathway [8]. This pathway has also been shown to be involved in controlling spindle polarity via the intracellular modulator LET-99 [28].

As a variation of the aforementioned mechanism of asymmetric spindle positioning can be considered the role of GPA-16 in the establishment of left-right (l-r) asymmetry. In *C. elegans*, l-r asymmetry first becomes apparent at the six-cell stage when the blastomeres ABal/ABpl are located more anteriorly than ABar/ABpr [29,30]. The mechanism underlying cell positioning is based on mitotic spindle orientation. In this context, it has been demonstrated that a temperature-sensitive *gpa-16* mutant displays a randomized ABa/p spindle skew handedness, suggesting that the Gα subunit is required for the positioning of ABa and ABp spindles preceding skewing [31]. The role of other G protein signaling components in this context as well as the downstream targets that execute spindle orientation remain to be clarified.

### 2.2. The GPCR Frizzled Is Involved in Polarity and Spindle Orientation 

The roles of several heterotrimeric G proteins in spindle orientation and cell division in early *C. elegans* embryogenesis has been firmly demonstrated in numerous studies (Section 2.1). However, these G-protein functions are independent of any GPCR and thus are termed “receptor-independent.” GPCRs are also associated with early embryonic cell division and polarity establishment. The most prominent GPCRs in this context are the four Frizzled family Wnt receptors, which control spindle polarity in the fourth round of cell division. In nematodes mutant for components of the Wnt pathway, spindles are tilted compared to those of wild-type embryos leading to severe defects, which in part also result in embryonic lethality [32,33,34,35,36]. The Wnt signaling pathway is required for anterior–posterior fate decisions. It polarizes the EMS blastomere for asymmetric cell division by conveying a signal from the neighboring P_2_ blastomere [34,35,37,38] (Figure 2B). Thereby, the cue produced by P_2_ is the Wnt ligand MOM-2 and the Frizzled homolog is MOM-5 [39]. The signals mediated by Wnt/Frizzled in this context involve components of the canonical Wnt signaling pathway, which is realized by several downstream molecules including Disheveled and β-catenin homologs (reviewed in [40,41], for some mechanistic details, see Section 2). However, to date no G proteins have been found to act in this context. 

Further, Wnt receptors are involved in other developmental processes, e.g., vulva and larval development, in which they also signal G protein-independently via a canonical pathway (summarized in [42]).

### 2.3. The GPCR LAT-1 Signals via G Proteins in Oriented Cell Division

As discussed above, GPCRs and G proteins are essential players in controlling spindle positioning and oriented cell division. However, in all these cases both molecules act independently of each other. Despite clear indications, GPCR-dependent and G protein-mediated signaling pathways had not been unambiguously defined for a long time [43,44]. Recently, the Adhesion GPCR Latrophilin-1 (LAT-1) has been identified to mediate spindle orientation in an anterior–posterior direction in distinct blastomeres from the 12-cell stage during symmetric cell division. This GPCR signals via the Gα_s_ protein GSA-1, ultimately increasing intracellular cAMP levels [45,46,47] (Figure 2C). Mutants null for *lat-1* display skewed division angles of the ABal blastomere (and some daughter cells), in a way that this division plane is almost perpendicular to MS, whereas in wild-type embryos the cell divides in an anterior–posterior direction. Although the details of the underlying mechanism such as whether it is a permissive or an instructive signal, the cell on which the signal is localized and the identification of other pathway components are yet to be clarified. This example shows that classical GPCR/G protein-mediated pathways are present in *C. elegans* development.

### 2.4. GPCRs and G Proteins in Patterning and Induction of the Vulva

GPCRs and G proteins also play a role in *C. elegans* development beyond early cell cleavages. One process in which both exert functions is the development of the vulva. The vulva of a hermaphrodite connects the uterus to the surrounding environment. It is formed from ventral epidermal precursors during larval development [30]. Besides Wnt/Frizzled signaling, which plays several roles during vulval development, including signaling through the HOX gene *lin-39* to generate six epidermal precursor cells and cell polarity [48,49], the Gα_q_ protein EGL-30 positively affects vulva development [50]. Whether this G protein is activated by a GPCR remains to be clarified. However, there is evidence that it might act in parallel to RAS and involve Wnt signals [50]. Further, the Gα protein GPA-5, which shares some homology with mammalian Gα_i_ proteins, negatively affects vulval development upon classical activation of the GPCR SRA-13 and by affecting RAS/MAPK signaling [51]. The site of action in this case is not determined, but the effect might be cell autonomous or stem from sensory neurons [51]. Further, large-scale RNAi screens have also revealed roles for GOA-1 in vulval development [52,53]. 

### 2.5. GPCRs and G Proteins in Neuronal Development

Neuronal migration is another process in postembryonic development that has been demonstrated to engage G proteins as well as GPCRs. Several lines of evidence point toward a role for the G proteins GOA-1 and EGL-30, as well as the GPCR Flamingo (FMI-1) in the migration of different neurons. GOA-1 is a serotonin effector in migrating neurons, with the N-type calcium channel homolog UNC-2 being a target for this signal [54]. Further, gain-of-function mutants of *egl-30* display defects in neuronal cell migration [54]. Whether these are GPCR-dependent functions remains to be determined. However, there is a GPCR known to be involved in neuronal cell migration: the Adhesion-GPCR FMI-1, a Flamingo/CELSR homolog that controls axon guidance. Loss-of-function alleles of *fmi-1* causes axon navigation defects of pioneer and follower axons in the ventral nerve cord [55]. The signal transduction and the molecular details of this receptor remains to be determined, but it has so far not been linked to any G protein activation.

### 2.6. Further Roles of G Proteins

Besides the relatively well understood processes discussed above, in which GPCRs and G proteins mediate functions in development, several studies point toward additional, yet not well understood, roles of G proteins. Whether these are dependent or independent of GPCRs will be interesting to be determined. For instance, there is some indication that GPB-1 appears to be involved in germline development. A *gpb-1* loss-of-function mutant displays, besides the defects during cell division in early embryonic development, abnormalities at later stages [24]. Some adults rescued by a *gpb-1* transgene in a mosaic manner are sterile and have abnormal germlines [24]. 

Considering their broad involvement in a plethora of functions, it is likely that many more of the still unknown functions of GPCRs and G proteins will be uncovered in the years to come.

In summary, it can be noted that GPCRs and G proteins in *C. elegans* development do act as both a functional unit and independently of each other, with the latter being a major part of early development, while classical G-protein function is more often found in later developmental stages. One possible reason for this could be the expansion of potential cellular contacts, which ask for increased communication and coordination. Future investigations will need to focus on the details of canonical and atypical G-protein functions, such as additional pathway components and especially their physiological impact. 

## 3. G Protein-Independent Function of GPCRs 

The main G protein-independent function of GPCRs in various aspects of *C. elegans* embryonic and larval development is the Wnt/Frizzled signaling pathway. A canonical versus a non-canonical one can be discriminated, which involve partially overlapping effector molecules. These pathways are conserved among species; however, in *C. elegans* cascades involving Wnt homologs and their receptors, which are slightly different from the ones found in mammals or *Drosophila melanogaster*, have been described. The details are summarized and discussed in [40,41]. The Wnt pathway controlling cell division and polarization of the EMS blastomere is mostly referred to as non-canoncial despite involving β-catenin (WRM-1). It is somewhat similar to the canonical pathway but also entails asymmetric localization of different components as a general mechanism. Downstream of the GPCR MOM-5, a Frizzled homolog binding the Wnt ligand MOM-2, key molecules such as the Disheveled homologs MIG-5 and DSH-2 and, subsequently, WRM-1 (Figure 2B) are recruited. Further mechanistic details of the different Wnt signaling pathways have been dissected in several studies and are described elsewhere (reviewed in [40,41]). While in other organisms it has been shown that the Wnt receptors Frizzled can act as classical GPCRs coupling to G proteins (reviewed in [56]), this information is lacking for *C. elegans*. However, it will be intriguing to see whether there is an intersection of the Wnt cascade with G proteins.

## 4. Mechanisms of GPCR-Independent Functions of Heterotrimeric G Proteins

Although it becomes increasingly clear that, during *C. elegans* development, several GPCR-independent functions of G proteins are key modulators in various processes, the control of spindle positioning in asymmetric cell division via GOA-1 and GPA-16 is by far the best understood. It is one key example of how G proteins are regulated by several modulators and what mechanisms are underlying this process.

For a long time, the common belief was that G proteins are activated solely by GPCRs and only convey signals received by an extracellular cue into cells. In the mid-1990s, it became clear that G proteins can be regulated; thus, signaling can be fine-tuned by various accessory proteins such as GDIs or GTPase-activating proteins (GAPs). Both inhibit the activity of the Gα subunit and consequently inactivate the G protein, but through different mechanisms: GDIs prohibit the exchange of GDP for GTP, whereas GAPs, which are also called regulators of G protein signaling (RGS), enhance the GTPase activity of Gα subunits. RGS proteins contain a name-coining RGS domain. While first described in yeast (reviewed in [57]), they are present in many species. The *C. elegans* genome encodes more than 20 proteins with one or more RGS domains [58]. Although not for all of them, the Gα subunit they regulate has been identified, RGS proteins play extensive roles in the nervous system and influence behaviour, chemosensation, and egg laying. 

The only RGS protein which has been beyond doubt identified to be involved in controlling G protein activity during development is RGS-7, which regulates GOA-1 in spindle positioning [13,59]. Loss of *rgs-7* leads to increased Gα activity and subsequently to a hyper-asymmetric cell division due to reduced pulling forces on the anterior pole of the dividing cell [59]. RGS-7 function is complemented by two other accessory proteins: the GDIs GPR-1 and GPR-2. Both show homologies to the Pins (partner of insecutable) from *Drosophila melanogaster*. Similar to the Pins, they contain a GoLoco/GPR motif through which they are thought to associate with the G protein and inhibit dissociation of GDP when binding to GOA-1 [13,14,23]. Similarly to depletion of the G protein [7], loss of GPR-1/GPR-2 leads to almost no spindle movement due to a lack of pulling forces [13]. Together with LIN-5 they form the ternary complex. 

Interestingly, the G protein cycle regulated by RGS-7 and GPR-1/2 is not activated by a GPCR. In this receptor-independent pathway, GOA-1/GPA-16, bound to the Gβ subunit GPB-1 and the Gγ subunit GPC-2 [7], are activated by a cytoplasmic GEF. Discovery of these non-receptor GEFs came as a surprise, but they are now established in many different species [60,61]. The receptor-independent GEF controlling activation of the Gα_o_ during early cell division in *C. elegans* is RIC-8 (Synembrin). This widely known GEF, which has also been shown to function in neurons [62] and potentially regulates the Gα_q_ EGL-30 [63,64], functionally couples to Gα_o_ during the first divisions of the *C. elegans* embryo [16,23,62]. RIC-8 can physically interact with both GOA-1 and GPA-16 and has been shown to act as a GEF for GOA-1 [16,23]. Loss of ric-8 leads to slower nuclear migration, decreased pulling forces, and overall embryonic lethality [23,62]. Even though RIC-8 function has been characterized in great detail, not only in *C. elegans* but also in *Drosophila* and mice [65,66], no binding motif for the G protein interaction has been identified to date. 

That a non-receptor GEF rather than a GPCR serves as an activator in the case of G protein-controlled asymmetric cell division at a very early embryonic stage is a conceivable concept. Especially so, as in the two-cell-stage embryo, there is not much communication with the environment due to the egg being in a shell-confined space. Although details of the cues triggering the spindle positioning are not the focus of this review, it is interesting to note that this receptor-independent function of heterotrimeric G proteins to control spindle positioning during cell division is not unique to *C. elegans*. There is evidence from several studies starting to surface suggesting that it is a more general mechanism, which is highly conserved. The ternary complex comprising the G protein, GPR-1/2, and LIN-5, which is required for correct spindle orientation and functions to anchor and localize GPR-1/2 [7,15], has the counterpart Gα_i_/LGN/NuMA in mammals that is also essential for spindle positioning [67,68,69]. Further, it was shown in HeLa cells that the GEF RIC-8, the homolog of *C. elegans ric-8*, seems to be involved in this process [69]. 

As discussed above, the G protein cycle of the receptor-independent function of the Gα_o_ proteins GOA-1 and GPA-16 is completed by RGS-7, which harbors an intrinsic GTPase activity. However, it is puzzling that, while GAPs normally inactivate G proteins, in this case the opposite is true [59]. These data raise the question of the relevance of GTP hydrolysis in the receptor-independent G-protein function. There is some evidence that indeed GTPase activity is required. Firstly, direct anchoring of microtubules to the cortex via dynein is insufficient for cell division [70], indicating that the anchoring apparatus is not just a simple attachment and that pulling force generation is a critical part of its function. This issue was resolved by identifying LIN-5 as the main regulator of force generation within the anchoring complex, suggesting that no further function is expected of the G protein [70]. Secondly, it is known that, in the ternary complex, GOA-1 and GPA-16, respectively, are interacting with GPR-1/2, which are GDIs and therefore depend on the GDP-bound state to stably interact with the G protein [71]. Furthermore, GPB-1 and GPC-2 act as a competitor of GPR-1/2, as GPB-1 is also a GDI and thus also binds Gα. This is supported by the observation that loss-of-function mutations of *gpb-1* or *gpc-2* result in increased pulling force-phenotypes in *C. elegans* embryos [8,23,26]. Lastly, it has been shown that cortical enrichment of RGS-7 positively regulates spindle movement [70], which is conceivable in the context of the GDP-bound form of the Gα subunit being the point of connection of the ternary complex to the membrane (Figure 2A). Several models have been suggested describing GOA-1 activation and function. However, no model exists that accommodates all the findings on this G protein. The classical G protein cycle certainly does not explain the GOA-1 function. For instance, it has been proposed that the active unit mediating pulling force is the Gα-GDP/GPR complex, as GOA-1 and GPR-1/2 positively modulate this process [13,14,23]. However, it cannot be ignored that Gα-GTP is the active form, since the GEF RIC-8 also positively influences force generation [15]. Thus, further analyses are required to obtain a more refined model, which also accomodates the role of GPA-16.

Together, while this data suggests that GTPase activity is relevant for G protein function in early *C. elegans* development, one key experiment shows that this does not seem to be entirely true. Fielmich et al. showed that spindle positioning and cortical pull are not significantly compromised when both GPR-1/2 and the Gα protein are replaced by an arbitrary membrane anchor [70]. This indicates that, indeed, the basis of G-protein function in the zygote might be GTPase-independent membrane anchoring. Nevertheless, as the G protein can act as a switch between an active and inactive state, it is obvious that throughout evolution this system has evolved to be regulated by various mechanisms (stated above), stressing the need for flexibility and fine tuning during spindle alignment and cell division. It would be interesting to search for regulators of other GTPases with their main role in structure and scaffolding, such as tubulin.

## 5. The Relationship of GPCRs and G Proteins over Time

The concept of GPCRs and G proteins being able to function together as well as separately renders a versatile system to control cellular processes and a plethora of functional possibilities. While in *C. elegans* all three combinations, GPCR/G-protein function, GPCR-independent G-protein function, and G protein-independent GPCR function, can be extensively found during embryonic development, other species also make use of them. For instance, the Wnt receptor Frizzled in mammals can couple to G proteins but also act independently (for a summary, see [56]). The effectors and modulators such as non-receptor GEFs, GAPs, or RGS are highly conserved (see Section 4) during evolution, suggesting that the independent system has not just developed recently. A closer look at evolutionary aspects of GPCRs and G protein manifests this notion.

### 5.1. Origin of the GPCR System

G protein and GPCR functions are some of the oldest signaling mechanisms observed to date with strong conservation among a multitude of species and, based on the prevalence of their precursor genes in both prokaryotes and eukaryotes, likely originated from one universal common ancestor (UCA) [72,73,74]. Still, since there are species containing only GPCRs or G proteins, it is clear that the two do not always have to function together but have also developed independent signaling pathways [60]. Furthermore, in species in which GPCR-dependent G protein signaling is observed, there is considerable variation in the number of pathway components [60]. This observation was previously explained by the system being highly adaptable and modulative, increasing and sometimes decreasing in complexity over time [60,72,75]. Even though unicellular organisms do sense their environment and have mechanisms of signal perception [76,77,78], there was still a need for more diverse and intricate signaling mechanisms during development of multicellularity [79,80]. This was especially the case as metazoans were faced not just with receiving cues from their environment but also with the task of coordinating the development, structure, and function of tissues and organs. These circumstances explain the dramatic expansions of different signaling systems, such as the receptor tyrosine kinases [81,82,83] and the GPCR system, especially in metazoans [60,72,84].

### 5.2. Origin of G Proteins

G proteins are descendants of the extended clade of Ras-like GTPases, which most likely underwent a split in prokaryotes, giving rise to the ancestors of small G proteins (the Ran-Ras-Rho-Rab-like group) and of Gα subunits (the MglA-Arf-Gα group), with the latter originally being involved in membrane trafficking. Interestingly, only later in evolution they were recruited to act as downstream signaling molecues of membrane receptors [85,86,87,88,89]. The ancestral Gα as well as the classical G protein cycle are likely to have originated in the last eukaryotic common ancestor (LECA) [60,90]. However, all human Gα subunits (Gs, Gi/o, Gq, and G12/13, as well as the Gv type—which is constrained to marine animals and some insects), two precursors of Gβ, and one of Gγ can only be dated back to the common ancestor of holozoans [91,92]. 

**Figure 3:**
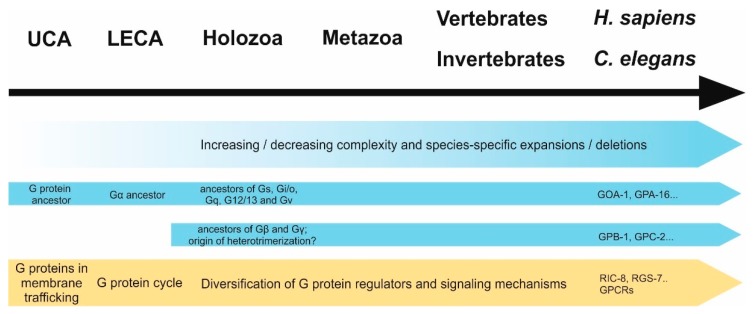
Development of G proteins (blue) and their functions (yellow) over time. First ancestors of G proteins and GPCRs have likely arisen in the universal common ancestor (UCA) of eukaryotes and prokaryotes, whereafter both underwent dramatic expansion and diversification. While the first appearance of a Gα protein is thought to be in the last common eukaryotic ancestor (LECA), precursor proteins of human Gα families as well as Gβ and Gγ subunits seem to have diverted in holozoans, suggesting that Gα could have had β/γ-independent functions pre-dating heterotrimerization. Before G proteins were recruited as downstream signals of membrane-bound receptors, they possibly functioned in membrane trafficking. Over time, not only their regulation through GEFs (such as GPCRs) but also other regulatory proteins (GAPs, GDIs) underwent expansion and gave rise to the multitude of G proteins signaling mechanisms we observe today.

In the same way as GPCRs, heterotrimeric G proteins have expanded to a great extent in some eukaryotic species [60,72,91], which was accompanied by strong diversification in genetic sequences (e.g., human and plant Gα subunits have approximately 33% similarity). Still, the crystal structures remain nearly identical [90], indicating conserved functions while also enabling different interactions due to sequential differences. On the other hand, there are eukaryotic clades—such as alveolates, kinetoplastids, and diplomonads—in which no evidence for heterotrimeric G proteins can be found to date [72]. A summary of the development of G proteins and their functions over time is shown in Figure 3.

In general, genes for GPCRs have expanded and undergone variation in higher frequency compared to G protein genes, leading to them having a smaller number of orthologs in more distant species [84]. However, many invertebrates including *C. elegans* underwent species-specific expansions of G proteins, even to a greater extent than, for example, humans [91,93], which could be a sign of acquisition of GPCR-independent pathways [90]. *C. elegans* in particular is truly interesting in this manner, since it harbors almost double the number of G proteins and GPCRs compared to humans [61], both of which have species-specific representatives that do not seem to be related to other species. In the case of GPCRs and many of the G proteins, this could be due to the extreme development of the chemosensory pathways of the nematode [6,94,95,96].

### 5.3. Atypical G-Protein Functions across Species

It is not easy to discuss whether GPCR-independent functions of G proteins existed before GPCR-dependent ones, as even though the two protein families appear to have emerged independently, the origins of both lay as far back in evolution as we are able to peek into (see above). However, most signaling pathways are composed of a rigid set of cytoplasmic elements, which can be modulated by various cues, and are transduced by a highly dynamic set of receptors [83,97,98]. This allows us to hypothesize the core signaling system (e.g., heterotrimeric G proteins and their respective downstram effectors) as a receptor-independent machinery that autonomously fulfills essential functions of the cell and ultimately constraints evolution. At the same time, receptors are present to modulate these functions and over the course of time develop different regulatory mechanisms (GEF/GAP), depending on what regulation is needed for the current state of G protein activity [90]. 

There is some data that supports this hypothesis: First of all, some evolutionary clades—such as embryophytes or unicellular holozoans—have been described to have no detectable or a reduced number of GPCRs or G proteins, respectively [60,91], suggesting that, indeed, they can function independently of each other. In the case of GPCRs, it is most likely that they use different downstream effectors (e.g., arrestins) and in the case of G proteins different regulators such as non-receptor GEFs, GDIs, and GAPs (addressed above). However, it has to be noted that the sole lack of a gene family is not necessarily a sign for an independent evolutionary origin, as loss during evolution can also be a result of irrelevance. For instance, transmembrane receptors are dispensable in the case of intracellular parasitism. In analogy to the lack of GPCRs, there are some species deficient of entire sets of G protein regulatory networks. For instance, in the early-branching eukaryotes such as *Trichomonas* and *Cyanophora paradoxa*, no β or γ subunits could be identified to date [60,72], suggesting that Gα acts independently of the other subunits. These data could further implicate that Gα subunits functioned on their own at first and only later partnered with Gβγ complexes. This hypothesis is consistent with the fact that Gα proteins seem to have emerged earlier in evolution (Figure 3).

However, the incredible variety of possible genetic constitutions of G protein signaling systems in different species does not entirely clarify the question of which of the regulatory elements arose first, but rather underlines the extreme modularity and flexibility of this network. Such flexibility allowed for classical and atypical functions to evolve independently, probably with many pathways diverging and converging throughout evolution. Thus, one challenge of future analyses on the entire spectrum of G-protein functions throughout the tree of life is to explain their evolutionary origin.

## 6. Tools for Studying GPCRs and G Proteins in *C. elegans*

Future studies need to focus on elucidating the mechanistic details of GPCR and G-protein function separately and together as well as gaining further insights into understanding their impact on development. Several tools have been developed in recent years to facilitate these research endeavors.

### 6.1. GPCR- and G Protein-Based Methods for Studying C. elegans Development

**Figure 4:**
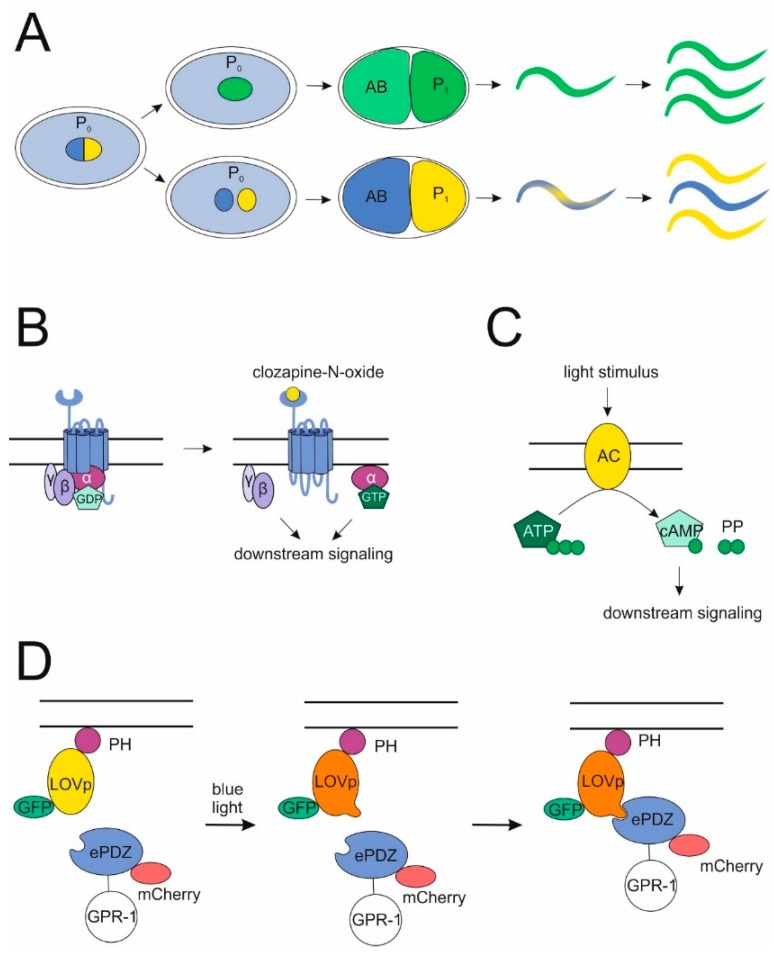
Novel methods for studying G-protein functions. (**A**) Non-mendelian genetic system. By overexpressing *gpr-1* in the *C. elegans* zygote instead of one bipolar spindle, two unipolar spindles are created, which impeaches pronuclear fusion and leads to chimeric embryos. Progeny of these can be genetic replicates of either the mother or the father, depending on which pronucleus localized to the P_1_ cell. (**B**,**C**) Specific induction of G-protein signaling. Distinct G protein cascades can be activated either by stimulating a DREADD, which cannot bind its cognate agonist anymore, with the inert drug clozapine-N-oxide (**B**) or by directly activating downstream effectors of certain G proteins such as an adenylyl-cyclase (downstream of a Gs protein) via photoinduction (**C**). (**D**) The PDZ-LOV-system. Exposure to blue light enables PDZ-tagged cytoplasmic proteins (such as GPR-1) to bind to LOV-domains, which are in parallel tethered to the membrane via a PH-anchor. Fluorescent molecules allow for the control of proper localization.

Even though many details of non-canonical functions of heterotrimeric G proteins in *C. elegans* development remain unclear, a solid foundation of knowledge is now established allowing for manipulations of known regulatory elements. The aim is to create new methods and model systems for developmental or general *C. elegans* research. One recent breakthrough was the establishment of a non-mendelian genetics system [99] (Figure 4A). This system is based on overexpression of the pulling force regulator *gpr-1*, which creates two unipolar spindles rather than a bipolar one in the *C. elegans* zygote. This impairs fusion of maternal and parental pronuclei, ultimately leading to genetically different AB and P_1_ blastomeres. One of them carries only maternal chromosomes, while the other inherits the parental counterparts. It remains unclear how the sets of chromosomes achieve diploidity, yet somehow about 30% of embryos survive to form lineage-specific hybrid nematodes and, as germ cells are descendants of the P_1_-lineage, the F2 generation is genetically identical to one of the parents, depending on which pronucleus was pulled to P_1_. This model can be used as a toolbox for many different approaches. The authors already described the relevance of parental/maternal hybrid animals as a means to investigate epigenetics, lineage-specific knockouts and mitochondrial or gonosomal gene transmission. It is conceivable that these model strains can also be employed in developmental research. For instance, now it is possible to easily create embryos which lack the ability to produce cues for cell division (in the style of the Wnt signal, that the P_2_ cell inflicts onto the EMS) to investigate the necessary communication between neighboring cells during development. In this way, not only necessity but also the directionality of signals between blastomeres can be thoroughly investigated. Furthermore, in tissues which are composed of both AB and P_1_ descendants such as the hypodermis, it could be possible to distinguish between genes required in every single cell of the tissue and genes, whose mere diffuse presence in the tissue is relevant.

Further, designer receptors and inducible downstream effectors can be used to study the impact of a GPCR or a G protein signaling pathway. Designer receptors exclusively activated by designer drugs (DREADDs) have been developed for the selective control of signaling in vivo in several organisms [100,101,102]. These modified muscarinic acetylcholine receptors are activated by the inert drug clozapine-N-oxide, but not by their endogenous agonists carbachol or acetylcholine. A DREADD activating the Gq signaling cascade has recently been developed for use in *C. elegans* [103] (Figure 4B). Another way to stimulate GPCR pathways is the use of photoactivatable cyclases producing cyclic AMP [104,105], which is a second messenger downstream of Gs-coupled GPCRs (Figure 4C). Both techniques could be used to selectively activate potential GPCR pathways to gain insights into their role in development.

### 6.2. Novel Methods Used to Investigate Atypical G-Protein Function

Several techniques potentially useful for G protein research in *C. elegans* have been introduced in the past years. An optogenetic system using a PDZ-tag on cytosolic proteins in combination with a membrane bound PH::LOV fusion protein was developed to selectively upregulate the surface expression of relevant proteins in the early embryo by exposure to blue light [70] (Figure 4D). One intriguing application of this technique was the interchangeability of G proteins in the early blastomeres by the PH::LOV-anchor, which solidifies the working model that GOA-1 and GPA-16 can fulfill their functions independent of their GTPase activity. However, this method harbors even more potential as it enables the selective upregulation of different regulators of cell division and cortical force generation in both wild-type and various mutant backgrounds, to characterize the effects on single cells as well as on the development of the entire embryo. It would also be interesting to express proteins ectopically at the cell surface to investigate their effects on the wild-type machinery.

In contrast to selective upregulation of protein expression, a recently developed method employs cell specific knockdown of mRNA by nonsense-mediated decay (NMD) [106]. In this system, a gene fusion was generated containing a gene of interest and part of the 3’-UTR of let-858, which is known to induce NMD on upstream genes [107], as their physiological stop codons are being recognized as premature. Secondly, a NMD-rescuing construct was designed, coding for one of the NMD machinery proteins (SMG-5), under the influence of a cell-specific promoter. 

Coexpression of these two in nematodes lacking both *smg-5* and the protein of interest leads to the first transgene rescuing gene activity in all cells except the ones defined by the promotor of the second transgene, ultimately leading to cell-specific knockdown. This system was used to show that GOA-1 has a specific function in HSN neurons controlling egg laying behavior, but we further propose the possibility of using blastomere-specific promoters to investigate functions of developmentally relevant proteins such as G proteins or G protein regulators in single blastomeres. Unfortunately, since active transcription only starts at the 8- to 12-cell stage [108], earlier blastomeres cannot be affected in this way. However, as stated above, it is possible to create embryos with genetically different AB and P_1_ cells by *gpr-1* overexpression, so that at early stages blastomere-specific knockdown could be achieved by mating mutant and wild-type parents and then generating hybrid offspring. These methods have the potential to increase our knowledge of spatio-temporal requirements of different proteins throughout development.

## 7. Conclusions and Outlook

The paradigm that GPCRs exclusively activate G proteins and that these heterotrimeric units act solely as signal transducers for the receptors has been disproven in the past decades by the discovery of GPCR effectors such as arrestins as well as different G protein regulators. We are just starting to begin to understand the principles as well as the relevance of this mutual infidelity. *C. elegans* development, especially embryonic development, is a prime example in which the density of GPCRs and G-protein function is high. However, the two do not necessarily act together. We can learn about how the GPCR/G protein system works in different contexts by looking at *C. elegans* development. For instance, the role of G proteins in left–right asymmetry in *C. elegans* embryos can offer a foundation for the understanding of some basic mechanisms in cardiogenesis. Overexpression of Gα during cardiogenesis in mice causes cardiac contractile failure [109] and left–right-handedness might be essential in this pathology. Further, the loss of the GEF Ric8A in mouse B cells causes a severe B cell immunodeficiency, likely due to reduced Gα_i_ protein activity and potential reduction in asymmetric cell division events [110]. Although the underlying mechanisms to establish left–right asymmetry are likely not entirely the same in mammals and *C. elegans*, knowledge of the role of G proteins or regulators such as RIC-8 in *C. elegans* can provide invaluable information to further understand and elucidate some of these processes in mammals. This knowledge transfer from basic principles to pathologies will be one major challenge in the future.

Understanding the GPCR/G protein mechanisms will be tremendously helpful in gaining insights into developmental processes. The question of why GPCR as well as separate G-protein functions mediate similar processes in spindle positioning and polarity during early cell division is highly intriguing, as are the potential GAP-independent G-protein functions. Further, there is evidence for several additional, yet undescribed G-protein functions in *C. elegans*. For instance, the presence of the GEF GBAS-1, which in other organisms preferably activates Gα monomers in contrast to GPCRs, which have higher affinity to heterotrimers (reviewed in [111]), has recently been shown [61].

Our understanding of GPCR and G-protein function in *C. elegans* is constantly growing and has yielded the development of a set of valuable tools to further investigate the signaling mechanisms and physiological concepts controlled by them and ultimately to answer the many remaining fundamental questions.
